# Loss of Beta‐Cell Identity and Function as a Mechanism of Secondary Failure of Sulfonylurea Therapy in Diabetes

**DOI:** 10.1002/mco2.70588

**Published:** 2026-02-09

**Authors:** Sumit Patel, Zihan Yan, Maria S. Remedi

**Affiliations:** ^1^ Department of Medicine Division of Endocrinology Metabolism and Lipid Research School of Medicine Washington University in St Louis St Louis Missouri USA; ^2^ Deparment of Cell Biology and Physiology School of Medicine Washington University in St Louis St Louis Missouri USA; ^3^ Center For the Investigation of Membrane Excitability Diseases School of Medicine Washington University in St Louis St Louis Missouri USA

**Keywords:** alpha‐cell, beta‐cell, diabetes, function, glucagon, insulin, identity, K_ATP_ channel, pancreas, sulfonylureas

## Abstract

Sulfonylureas, commonly used to treat type 2 diabetes (T2D), often lose effectiveness over time when used as monotherapy; however, the underlying mechanisms remain unclear. To investigate the mechanisms of sulfonylurea failure, glibenclamide‐releasing pellets were implanted in KK mice, a polygenic model that spontaneously develops T2D. KK mice receiving placebo pellets (KK‐Placebo) developed hyperglycemia, hyperinsulinemia, glucose intolerance, and insulin resistance. Notably, KK mice implanted with glibenclamide (KK‐Glib) showed improved blood glucose levels during the first 7 days, returning to KK‐Placebo levels thereafter. KK‐Glib mice exhibited reduced plasma insulin levels and insulin secretion in response to a glucose challenge compared with the markedly elevated levels in KK‐Placebo mice. KK‐Glib mice showed islet hypertrophy, reduced β‐cell mass and number, and increased α‐cell number, resulting in elevated α:β cell ratio compared with KK‐Placebo. Although mRNA expression of β‐cell identity markers remained unchanged, their protein levels were reduced in KK‐Glib, suggesting β‐cell identity loss, which may underlie the observed impaired insulin secretion. Remarkably, KK‐Glib mice showed elevated mRNA levels of *Ngn3* (dedifferentiation) and α‐cell identity markers along with glucagon content, suggesting α‐cell neogenesis. These findings suggest that secondary failure of sulfonylurea therapy may, in part, result from loss of β‐cell identity‐function and increased α‐cell number‐identity.

## 1 Introduction

According to the International Diabetes Federation approximately 537 million people worldwide were living with diabetes in 2021, with 90% of these cases being type 2 diabetes mellitus (T2D) [1]. T2D results from a gradual loss of functional pancreatic β‐cell mass, along with insulin resistance in peripheral tissues [2, 3]. Glycemic control remains a key focus in the management of patients with T2D. Biguanides (metformin), insulin sensitizers, alpha glucosidase inhibitors, incretin mimetics, amylin antagonists, sodium‐glucose cotransporter‐2 inhibitors, and insulin secretagogues remain common monotherapies or combination therapies for achieving and maintaining glycemic control in T2D [4].

Although the newer antidiabetic agents can be a preferable option for many individuals with diabetes, sulfonylureas (SUs) remain widely prescribed worldwide [5]. SUs have long been used to treat T2D because of their ability to inhibit ATP‐sensitive K^+^ (K_ATP_) channels, thereby stimulating insulin secretion, independently of the metabolic state of the cell. K_ATP_ channels in the pancreatic β‐cells are composed of four pore‐forming Kir6.2 (encoded by *Kcnj11*) and four regulatory sulfonylurea receptor SUR1 (ATP‐binding cassette encoded by *Abcc8*) subunits [6]. SUs such as glibenclamide/glyburide and tolbutamide bind to the SUR1 regulatory subunit of the K_ATP_ channel leading to their closure. This leads to plasma membrane depolarization, influx of Ca^2+^ through voltage‐gated Ca^2+^ channels, increase in intracellular [Ca^2+^], and the subsequent fusion of the insulin‐containing vesicles with the membrane, resulting in insulin secretion [6, 7].

Although initially effective, SUs exhibit shorter durability of glycemic control and higher rates of secondary failure than other antihyperglycemic agents [8, 9, 10], eventually losing their efficacy as monotherapy for reasons that remain unclear [11, 12, 13]. Early introduction of metformin in patients already receiving sulfonylurea therapy has been shown to improve glycemic control compared with sulfonylurea treatment alone [14]. However, the mechanisms underlying sulfonylurea failure and the progressive loss of drug responsiveness in humans remain elusive.

To investigate the mechanisms of sulfonylurea failure during diabetes progression in vivo, we used KK mice, an inbred strain established in the late 1950s and known for their spontaneous development of moderate diabetes and glucose intolerance [15, 16] progressively worsen over time. These metabolic abnormalities are accompanied by glucosuria, impaired insulin secretion, and insulin resistance, recapitulating many key features of T2D [, 17]. To determine the mechanisms underlying secondary sulfonylurea failure in diabetes, slow‐release glibenclamide pellets (2.5 mg/pellet, 60‐day release) were implanted in 6‐week‐old KK mouse model of polygenic T2D and follow over time.

## 2 Results

### 2.1 Chronic Glibenclamide Treatment Shows No Improvement in Blood Glucose Levels and Decreased Plasma Insulin Levels

**FIGURE 1:**
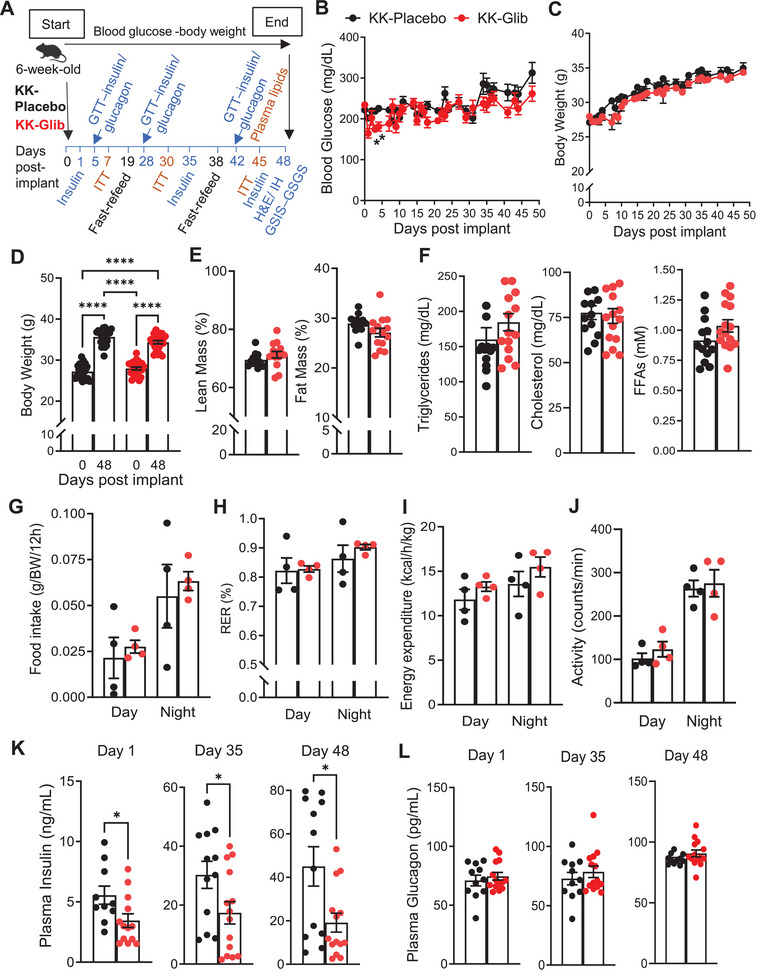
Chronic glibenclamide treatment shows no improvement in blood glucose levels and decreases plasma insulin levels in KK mouse model of T2D. (A) Experimental scheme to determine the effects of glibenclamide in KK mouse model of T2D. (B and C) Fed blood glucose levels and body weight monitored daily for the course of 48 days (*n *= 22–24 mice/group). (D) Body weight gain (*n *= 22–24 mice/group). (E) Lean and fat mass composition after 48 days of treatment (*n *= 12–14 mice/group). (F) Plasma triglycerides, cholesterol, and free fatty acids (FFAs) after a 4 h fast, 45 days posttreatment (*n *= 13–15 mice/group). (G) Food intake, (H) respiratory exchange ratio (RER), (I) heat/energy expenditure, and (J) physical activity/movement during dark and light cycles (*n *= 4 mice/group). (K) Fed plasma insulin levels and (L) fed plasma glucagon levels at 1‐, 35‐, and 48‐days posttreatment (*n *= 11–14 mice/group). Data are mean ± SEM. Statistical analysis was conducted by unpaired Student's *t*‐test or two‐way ANOVA followed by the posthoc Tukey's test as appropriate. *p < 0.05, ****p < 0.0001. Nonsignificant differences are not shown.

To determine the chronic effects of SUs in diabetes in vivo, we used polygenic KK mouse model of T2D, which develop hyperglycemia by 6 weeks of age and exhibit progressive increases in blood glucose levels over time [17]. At 6 weeks, KK mice were implanted with 60‐day slow‐release pellets to ensure sustained drug exposure over a chronic period to test the effect of the drug (glibenclamide, schematic timeline in Figure 1A). Pellets utilized contained either 2.5 mg glibenclamide, a dose commonly used in individuals with diabetes or placebo [18, 19]. KK mice implanted with glibenclamide pellets showed reduced fed blood glucose levels during the first 7 days postimplantation, with a significant decrease observed on Days 3 and 4 (Figure 1B). However, blood glucose levels subsequently returned to those observed in the KK‐Placebo‐implanted mice (Figure 1B). KK‐Glib mice did not exhibit significant changes in body weight over time compared with KK‐Placebo mice (Figure 1C), with both groups showing similar increase in weight gain over the 48‐day period (Figure 1D). Lean and fat mass composition did not differ between KK‐Glib and KK‐Placebo mice (Figure 1E). Additionally, the weights of the liver, epididymal white adipose tissue, and brown adipose tissue were comparable between the two groups (Figure S1A–C, respectively). Moreover, plasma levels of triglycerides, cholesterol, and free fatty acids (FFAs) did not differ significantly between groups (Figure 1F). Similarly, food intake was comparable between KK‐Glib and KK‐Placebo mice (Figure 1G). Respiratory exchange ratio (RER; Figure 1H), energy expenditure (Figure 1I), and physical activity (Figure 1J) remained unchanged between groups 48 days posttreatment. Strikingly, while fed plasma insulin levels were significantly reduced in KK‐Glib mice compared with the KK‐Placebo group on Days 1, 35, and 48 (Figure 1K), plasma glucagon levels did not differ between the two groups (Figure 1L).

### 2.2 Glibenclamide Does Not Improve Glucose Tolerance and Impairs Insulin Secretion

**FIGURE 2:**
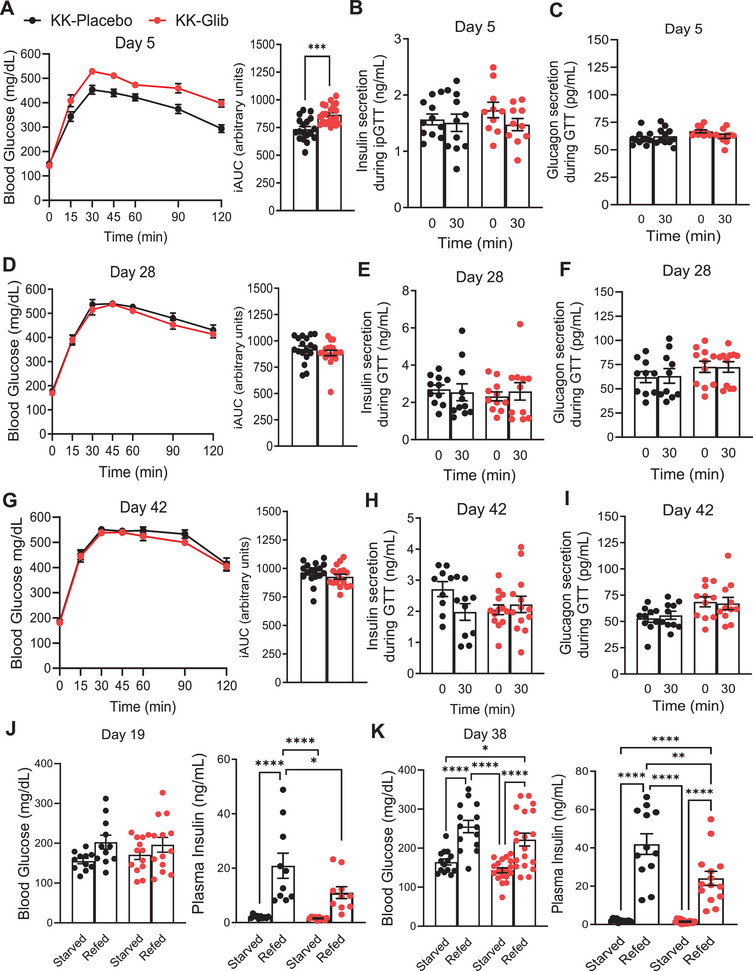
Chronic glibenclamide treatment shows no improvement in glucose tolerance and impairs insulin secretion in vivo. (A) Intraperitoneal glucose tolerance test (ipGTT) and incremental area under the curve (iAUC) performed Day 5 posttreatment (*n *= 18–21 mice/group). (B) Insulin secretion and (C) Glucagon secretion during the ipGTT Day 5 posttreatment at 0′ (fasted) and at 30′ min post‐i.p. glucose administration (*n *= 11 mice/group). (D) ipGTT and iAUC performed Day 28 posttreatment (*n *= 18–19 mice/group). (E) Insulin secretion and (F) glucagon secretion during the ipGTT Day 28 posttreatment at 0′ (fasted) and at 30′ min post‐i.p. glucose administration (*n *= 10–11 mice/group). *G* ipGTT and iAUC performed Day 42 posttreatment (*n *= 17 mice/group). (H) Insulin secretion and (I) glucagon secretion during the ipGTT Day 42 posttreatment at 0′ (fasted) and at 30′ min post‐i.p. glucose administration (*n *= 10–13 mice/group). (J) Blood glucose (*n *= 11–13 mice/group) and plasma insulin (*n *= 10 mice/group) levels after fasting‐refeeding at Day 19 posttreatment. *K* Blood glucose (*n *= 17–19 mice/group) and plasma insulin (*n *= 12–14 mice/group) levels after fasting‐refeeding Day 38 posttreatment. Data are mean ± SEM. Statistical analysis was conducted by unpaired Student's *t*‐test or two‐way ANOVA followed by the posthoc Tukey's test as appropriate. *p < 0.05, **p < 0.01, ***p < 0.001, ****p < 0.0001. Nonsignificant differences are not shown.

We next evaluated the effects of chronic glibenclamide treatment on insulin and glucagon secretion in response to a glucose challenge in vivo. KK mice implanted with glibenclamide pellets showed significantly impaired glucose tolerance with respect to the KK‐Placebo group at 5 days postimplantation (Figure 2A). Fasting plasma insulin and glucagon levels were similar between KK‐Placebo and KK‐Glib mice prior to glucose challenge, with no significant increase in plasma insulin or glucagon during the in vivo glucose challenge (time 0‐ and 30‐min postglucose injection; Figure 2B,C, respectively). Interestingly, KK‐Glib mice exhibited similar glucose tolerance, as well as fasting insulin and glucagon levels compared with KK‐Placebo mice at Day 28 post pellet implantation (Figure 2D–F), with no differences observed at 30 min after glucose challenge (Figure 2E,F). These effects persisted through Day 42 postimplantation (Figure 2G–I).

To determine the impact of nutrient availability on insulin secretion, we performed a fast‐refeed experiment on Days 19 and 38 postglibenclamide treatment. Fasting blood glucose and 2‐h postprandial blood glucose did not differ between KK‐Glib and KK‐Placebo mice on Day 19 (Figure 2J). While fasting insulin levels were similar between the two groups and they significantly increased in both groups after 2 h of refeeding, KK‐Glib mice demonstrated significantly lower levels of plasma insulin after refeeding compared with KK‐Placebo mice (Figure 2J). Body weights were unchanged either in fasting or after refeeding conditions on Day 19 (Figures S2A). Interestingly, while fasting blood glucose levels were similar between the KK‐Glib and KK‐Placebo mice, blood glucose was significantly higher postprandially in both groups, with no differences between the groups at Day 38 postpellet implantation (Figure 2K). Although fasting insulin levels were similar between the two groups at Day 38, and significantly elevated in both groups 2 h postprandially, the increase in KK‐Glib mice was again significantly lower compared with KK‐Placebo mice (Figure 2K). Body weights significantly decreased in KK‐Glib mice after 16 h of fasting compared with KK‐Placebo group, and although increased in both groups after 2 h of feeding (Figure S2B), the increase observed in KK‐Glib was significantly lower compared with KK‐Placebo at Day 38 postpellet implantation (Figure S2B).

### 2.3 Prolonged Glibenclamide Treatment Abrogates the Early Improvement in Insulin Sensitivity

**FIGURE 3:**
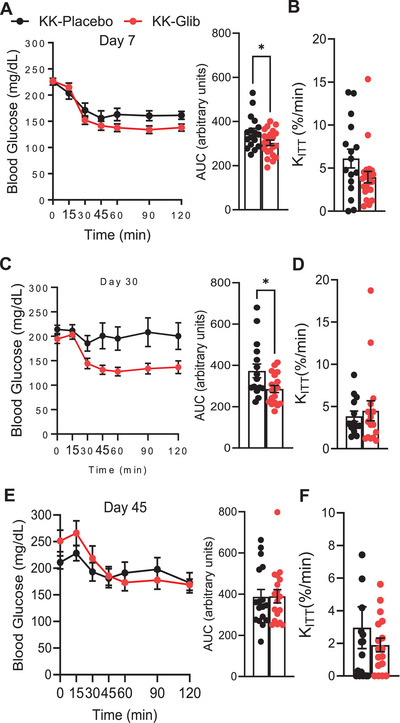
Chronic glibenclamide treatment improves insulin sensitivity in KK mice, but this effect is lost with long‐term treatment. (A) Intraperitoneal insulin tolerance test (ipITT) and area under the curve (AUC) and (B) *K*_ITT_ values performed at Day 7 posttreatment (*n *= 17–22 mice/group). (C) ipITT, AUC, and (D) *K*_ITT_ values performed at Day 30 posttreatment (*n *= 17–18 mice/group). (E) ipITT, AUC, and (F) *K*_ITT_ values performed at Day 45 posttreatment (*n *= 17–18 mice/group). Data are mean ± SEM. Statistical analysis was conducted by unpaired Student's *t*‐test. *p < 0.05. Nonsignificant differences are not shown.

We further evaluated the impact of glibenclamide on insulin sensitivity by performing insulin tolerance tests (ipITT). Insulin sensitivity was significantly improved in KK‐Glib mice compared with KK‐Placebo on Day 7 and 30 postimplantation (Figure 3A,C; area under the curve: AUC); however, this effect was abrogated at Day 45 of glibenclamide treatment (Figure 3E). Importantly, the plasma glucose disappearance rate (*K*_ITT_) in response to insulin did not differ between KK‐Glib and KK‐Placebo groups at Days 7, 30, or 45 postimplantation (Figure 3B,D,F, respectively).

### 2.4 Glibenclamide Decreases GSIS But Has No Effect on Glucose‐Stimulated Glucagon Secretion Ex Vivo

**FIGURE 4:**
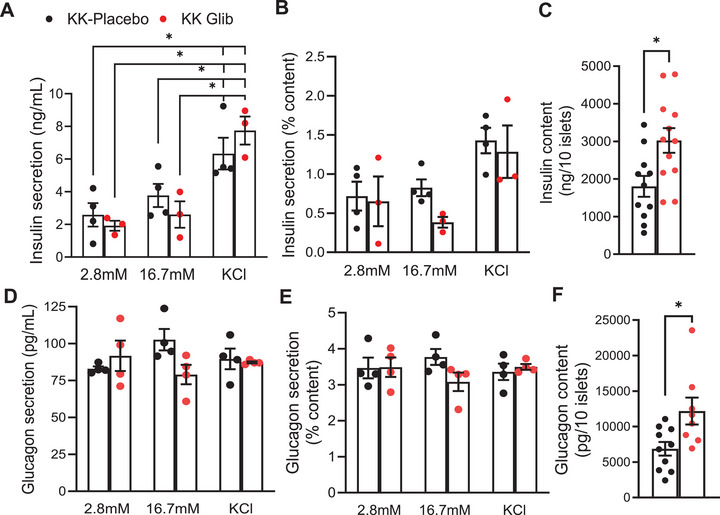
Chronic glibenclamide treatment decreases GSIS in KK mice. (A) Glucose‐stimulated insulin secretion (GSIS) at basal (2.8 mM) glucose, high (16.7 mM) glucose, or KCl (30 mM) from freshly isolated islets after 48 days postintervention (*n *= 3–4 mice/group). (B) GSIS from freshly isolated islets normalized to total insulin content (*n *= 3/4 mice/group). (C) Total insulin content from freshly isolated islets (*n* = 3–4 mice/group). (D) Glucose‐stimulated glucagon secretion (GSGS) at basal (2.8 mM) glucose, high (16.7 mM) glucose, or KCl (30 mM) from freshly isolated islets after 48 days of intervention (*n *= 3/4 mice/group). (E) GSGS from freshly isolated islets normalized to total glucagon content (*n* = 3–4 mice/group). (F) Total glucagon content from freshly isolated islets (*n *= 3–4 mice/group). Data are mean ± SEM. Statistical analysis was conducted by unpaired Student's *t*‐test or two‐way ANOVA followed by the posthoc Tukey's test as appropriate. *p < 0.05. Nonsignificant differences are not shown.

To further evaluate the effects of chronic glibenclamide treatment on β‐ and α‐cell function, we performed glucose‐stimulated insulin and glucagon secretion assays using freshly isolated islets from KK‐Glib and KK‐Placebo mice at Day 48 postimplantation, thereby avoiding potential drug washout effects. Islets from KK‐Glib mice showed no difference in insulin secretion compared with islets from KK‐Placebo mice when incubated at low glucose (2.8 mM; Figure 4A,B). However, insulin secretion in response to high glucose (16.7 mM) was slightly, but not significantly, reduced in KK‐Glib islets with respect to KK‐Placebo (*p* = 0.056; Figure 4A,B), correlating with results when insulin secretion was normalized to insulin content (Figure 4B), which was itself significantly elevated in KK‐Glib islets (Figure 2C). Both groups exhibited a similar increase in insulin secretion upon stimulation with 30 mM KCl (Figure 4A,B). Interestingly, insulin content was increased in islets from KK‐Glib mice compared with islets from KK‐Placebo mice (Figure 4C). Additionally, mRNA levels of *Epac2*, which regulate insulin secretion, and *Snap25*, a key marker of insulin exocytosis, remained unchanged between groups (Figure S2C). Islet glucagon secretion did not differ significantly in islets from KK‐Glib and KK‐Placebo mice under low glucose (2.8 mM), high glucose (16.7 mM) or KCl‐stimulated conditions (Figure 4D,E), but glucagon content was significantly increased in islets from KK‐Glib mice (Figure 4F). To assess the potential washout effect of glibenclamide, islets from KK‐Glib and KK‐Placebo mice were cultured overnight before measuring glucose‐ and KCl‐stimulated insulin secretion. After overnight culture, islets from KK‐Glib mice exhibited higher insulin secretion at both low and high glucose concentrations, as well as under KCl stimulation, compared with KK‐Placebo mice (Figure S2D). Importantly however, these differences disappeared when insulin secretion was normalized to insulin content (Figure S2E), which was itself significantly elevated in KK‐Glib islets (Figure S2F).

### 2.5 Chronic Glibenclamide Treatment Increases Islet Size, β‐Cell Proliferation, and α:Β Cell Ratio

**FIGURE 5:**
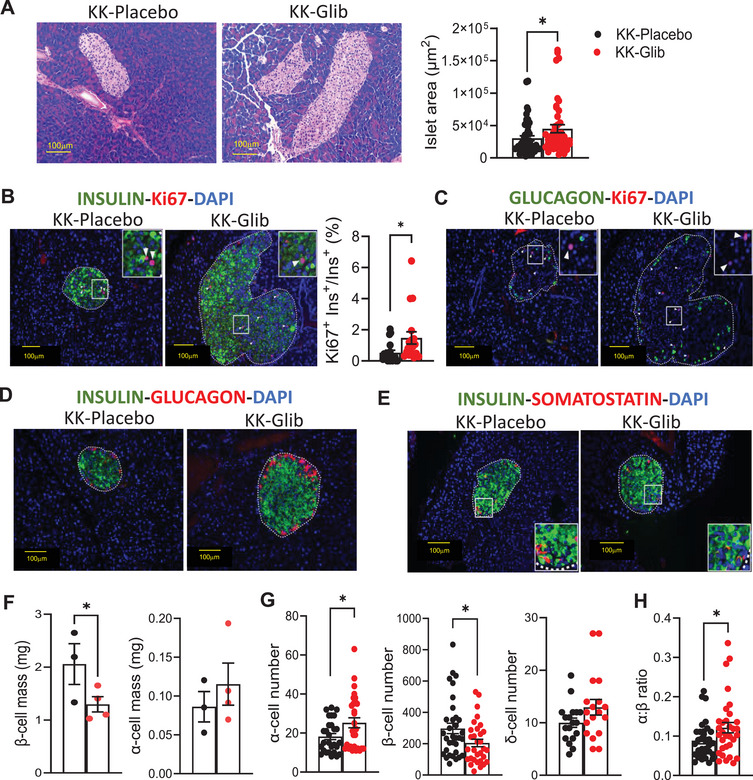
Increased islet area and α‐cell mass and number in KK‐Glib mice. (A) Representative images for H&E staining of pancreata (left, scale bars 100 µm, *n *= 4 mice/group) and quantification of islet area (*n *= 45–48 islets from 4 mice/group). (B) Representative images of immunofluorescent staining of Ki67 and insulin at Day 48 posttreatment (scale bars 100 µm, *n *= 3–4 mice/group) and quantification of Ki67^+^INS^+^(*n *= 18 islets from 4 mice/group), arrows indicate Ki67^+^ cells. (C) Representative images of immunofluorescent staining of Ki67 and glucagon at Day 48 posttreatment, arrows indicate Ki67^+^ cells. (D) Representative immunostaining images of insulin (green) and glucagon (red) staining in pancreata (scale bars 100 µm, *n *= 4 mice/group). (E) Representative immunostaining images of insulin (green) and somatostatin (red) staining in pancreata (scale bars 100 µm, *n *= 3 mice/group). (F) Quantification of β‐cell mass and α‐cell mass in islets from KK‐Glib and KK‐Placebo mice 48 days posttreatment (*n *= 3–4 mice/group). *G* Number of α‐ and β‐cells (*n *= 28–30 islets from 4 mice/group) and number of δ‐cells (*n *= 18 islets from 3 mice/group) in islets from KK‐Glib and KK‐Placebo mice. (H) Ratio of α‐cells to β‐cells (*n *= 32–33 islets from 4 mice/group) 48 days posttreatment. Statistical analysis was conducted by unpaired Student's *t*‐test. *p < 0.05. Nonsignificant differences are not shown.

Hematoxylin–eosin (H&E) staining of pancreatic sections demonstrates a significant increase in islet size in KK‐Glib mice compared with islets from KK‐Placebo mice (Figure 5A). To determine whether this increase was due to cell proliferation, we performed immunofluorescence for the nuclear proliferation marker Ki67 along with insulin or glucagon. The percentage of Ki67^+^insulin^+^ cells was significantly higher in islets from KK‐Glib mice compared with KK‐Placebo mice (Figure 5B, unmerged images in Figure S4C), whereas α‐cell proliferation remained unchanged (Figure 5C). Interestingly, immunostaining for glucagon and insulin showed increased insulin and glucagon in islets from KK‐Glib mice (Figure 5D), correlating with the significant increase in insulin and glucagon content observed in islets from these mice (Figure 4C,F, respectively). In contrast, somatostatin (Sst) immunostaining was similar in islets from KK‐Glib and KK‐Placebo mice (Figure 5E). Importantly, although α‐cell mass showed a slight but not significant increase in α‐cell mass, β‐cell mass was significantly reduced in islets from KK‐Glib mice compared with KK‐Placebo mice (Figure 5F). Strikingly, these results correlate with an increase number of α‐cells, a decreased in the number of β‐cells, and no change in the number of δ‐cells in islets from KK‐Glib mice compared with KK‐Placebo mice (Figure 5G, unmerged images in Figure S3A), contributing to the increased in α:β cell ratio observed in KK‐Glib mice (Figure 5H).

### 2.6 Chronic Treatment With Glibenclamide Decreased β‐Cell Identity and Increased Markers of α‐Cell Identity

**FIGURE 6:**
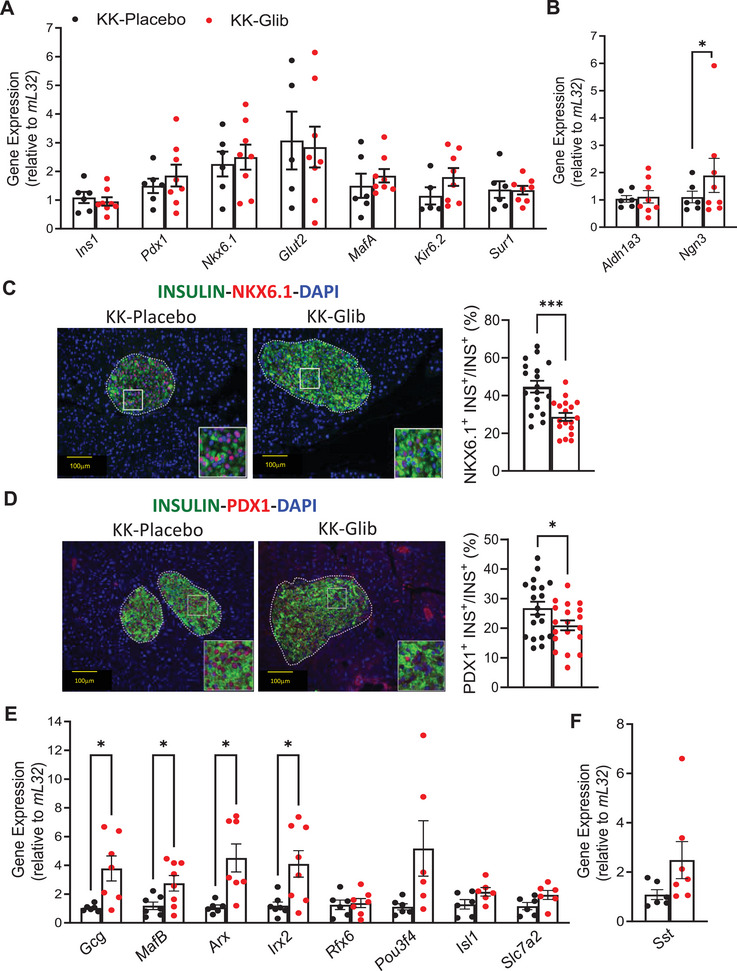
Chronic treatment with glibenclamide decreased β‐cell identity and increased markers of α‐cell identity. (A) Quantitative real‐time PCR analysis for mature β‐cell identity markers such as *Ins1*, *Pdx1*, *Nkx6.1*, *Glut2*, *MafA*, and *Kir6.2* and *Sur1* (*n *= 6–8 mice/group). (B) Quantitative real‐time PCR analysis for the dedifferentiation markers *Aldh1a3* and *Ngn3* (*n *= 6–8 mice/group). (C) Representative images of immunofluorescent staining of NKX6.1 (scale bars 100 µm, *n *= 4 mice/group) and quantification of NKX6.1^+^INS^+^ (*n *= 18 islets from 3 to 4 mice/group). (D) Representative images of immunofluorescent staining of PDX1 (scale bars 100 µm, *n *= 4 mice/group) and quantification of NKX6.1+INS+ (*n *= 18 islets from 3 to 4 mice/group). (E) Quantitative real‐time PCR analysis for the alpha cell markers *Gcg*, *MafB*, *Irx2*, *Rfx6*, *Pou3f4, Isl1*, and *Slc7a2* (*n *= 6–8 mice/group). (F) Quantitative real‐time PCR analysis in islets for *Sst* (*n *= 6–8 mice/group). Data are mean ± SEM. Statistical analysis was conducted by unpaired Student's *t*‐test. *p < 0.05, **p < 0.01, ***p < 0.001, ****p < 0.0001. Nonsignificant differences are not shown.

To test whether the changes in α‐ and β‐cells numbers were driven by changes in cell identity, islets and pancreases from KK‐Glib and KK‐Placebo mice were subjected to quantitative RT‐PCR or immunostaining, respectively. While gene expression of β‐cell identity markers such as *Ins1, Pdx1, Nkx6.1, Glut2*, and *MafA* did not significantly differ between KK‐Glib and KK‐Placebo mice (Figure 6A), immunostaining for both NKX6.1 and PDX1 proteins showed a significant reduction in KK‐Glib mice (Figure 6C,D, respectively, unmerged images in Figure S4A,B). Importantly, mRNA levels of the K_ATP_ channel subunits, *Kir6.2* and *Sur1*, were not significantly different between islets from KK‐Glib and KK‐Placebo mice (Figure 6A). While mRNA levels of *Aldh1a3*, a dedifferentiation marker, did not significantly change in islets from KK‐Glib and KK‐Placebo mice (Figure 6B), *Ngn3* mRNA levels, another marker of dedifferentiation to endocrine progenitor cells, significantly increased in islets from the KK‐Glib group (Figure 6B). Notably, markers of α‐cell identity such as *Gcg, MafB, Arx*, and *Irx2* were significantly increased in islets from KK‐Glib mice compared with islets from KK‐Placebo mice (Figure 6E). Moreover, *Pou3f4/Brn4* and *Slc7A2* genes that are predominantly expressed in α‐cells, also increased in islets from KK‐Glib mice (Figure 6E; *p* = 0.06 and *p* = 0.08, respectively). *Sst* mRNA levels did not change in islets from KK‐Glib compared with KK‐Placebo mice (Figures 6F and S3A,B), and, in agreement with this, δ‐cell number did not show any differences between KK‐Glib and KK‐Placebo groups (Figure 6F).

## 3 Discussion

T2D is characterized by insulin secretory deficiency in response to increased blood glucose levels. Several drugs have been developed to lower blood glucose, including SUs, which inhibit β‐cell K_ATP_ channels and stimulate insulin secretion. However, the effectiveness of SUs has been questioned due to secondary failure following prolonged use. In this study, we demonstrate that 48 days of glibenclamide treatment in polygenic diabetic KK mice impaired insulin secretion, which is driven by a complex interplay of loss of β‐cell identity and increased α‐cell number and identity. SUs such as glimepiride have been used in various rodent models, with studies reporting either a decrease [20, 21, 22, 23, 24, 25] or no changes [26, 27, 28] in blood glucose levels, which could partly be due to variations in the duration and dosage across studies. Our findings show a reduction of blood glucose levels within the first 7 days of treatment, followed by a rebound to KK‐Placebo levels thereafter (Figure 1B). The reduction of insulin output in a nonfasting state was evident as early as Day 1 postimplantation, with similar reduction observed on Days 35 and 48 (Figure 1K). These results suggest that acute and chronic glibenclamide treatment reduces insulin secretion from the already abnormally very high levels observed in placebo‐treated KK mice, rather than enhancing it (Figure 1K). These findings are consistent with studies reporting reduction in nonfasted plasma insulin levels in C57BL/6J mice treated with glimepiride or glibenclamide [29, 30]. Moreover, they correlate with earlier in vitro studies suggesting that loss of sulfonylurea‐induced insulin secretion may result from a decreased number of functional K_ATP_ channels on the plasma membrane, without affecting the transcription of the Kir6.2 or SUR1 subunits (Figure 6A) [31, 32, 33, 34, 35, 36]. Our results demonstrate that KK‐Glib exhibit impaired glucose tolerance on Day 5, despite no differences in fasting or glucose‐stimulated insulin secretion (GSIS) compared with KK‐Placebo mice (Figure 2A,B). This suggests that does nor further enhance the already markedly elevated insulin secretion observed in KK‐Placebo mice, consistent with previously reported abnormally high insulin levels in KK mice [17]. These findings also align with previous studies showing that high doses of SUs can impair glucose tolerance in various mouse strains [29, 30]. Moreover, glucagon secretion did not differ between KK‐Glib and KK‐Placebo mice under either fasting or stimulatory conditions (Figure 2C).

K_ATP_ channels are also expressed in α‐cells, with the same subunit composition as in the β‐cells, making them potential binding targets for SUs [37, 38]. The effect of SUs on glucagon secretion remains controversial, with some studies reporting an increase [39, 40, 41, 42, 43, 44, 45, 46, 47, 48], others a decrease [49, 50, 51, 52, 53], and some showing no effect [54, 55]. In our study, we did not observe significant differences in nonfasted glucagon levels between KK‐Glib and KK‐Placebo mice (Figure 1L). This is consistent with previous findings in C57BL/6J mice where glimepiride treatment did not alter serum glucagon levels compared with untreated controls [29]. Additionally, impaired glucagon secretion has been reported in mice lacking the SUR1 subunit of the K_ATP_ channels [56], and recent reports suggest that SUs may stimulate glucagon secretion by increasing intracellular [Ca^2+^] or by inhibiting it through a paracrine effect mediated by Sst [57]. In addition, SUs have been shown to suppress glucagon secretion during insulin‐induced hypoglycemia in both healthy individuals and those with T2D [58, 59]. In our study, KK‐Glib mice exhibited similar glucose tolerance as the KK‐Placebo mice, with no significant changes in insulin or glucagon levels, either in fasting or under glucose‐stimulation in vivo at both Day 28 (Figure 2D–F) and 42 postimplantation (Figure 2G–I). Following refeeding, both KK‐Glib and KK‐Placebo mice showed increased insulin secretion, however, the response was significantly blunted in KK‐Glib mice compared with KK‐Placebo mice at Days 19 and 38 of treatment (Figure 2J,K).

Interestingly, the improved insulin sensitivity in KK‐Glib mice on Days 7 (Figure 3A) and 30 (Figure 3C) parallels previous findings in glyburide‐treated Sprague–Dawley rats [60]. Our results are also consistent with reports of decreased Homeostasis Model Assessment‐estimated Insulin Resistance, indicative of improved insulin sensitivity, in diabetic albino Wistar rats treated with glibenclamide [61]. However, while glibenclamide therapy significantly improved insulin sensitivity in healthy human subjects, no such effect was observed in individuals with diabetes [62], which may parallel to the loss of insulin sensitivity observed in KK‐Glib mice by Day 45 (Figure 3E). Notably, insulin sensitivity remained unchanged in nondiabetic C57BL/6J mice implanted with either low or high doses of glibenclamide pellets [30], suggesting that the mechanisms regulating insulin responsiveness may differ between diabetic and nondiabetic states and in obese versus lean individuals. Nevertheless, *K*_iTT_ values did not differ between KK‐Glib and KK‐Placebo groups, as blood glucose declined at a similar rate in both groups over 0–30 min period following insulin administration (Figure 3B,D,F).

Furthermore, ex vivo GSIS in freshly isolated islets at Day 48 postimplantation showed reduced insulin secretion at high glucose in KK‐Glib mice compared with KK‐Placebo mice (Figure 4A). This reduction was more pronounced when expressed as a percentage of insulin content, which was significantly elevated in KK‐Glib islets (Figure 4B,C, respectively). No differences were observed in insulin secretion under low glucose or KCl‐stimulated conditions (Figure 4A,B). Glucagon secretion remained unchanged under low or high glucose or under KCl‐stimulated conditions (Figure 4D,E). The absence of changes in *Epac2* and *Snap25* mRNA levels in islets from KK‐Glib mice (Figure S2C) suggests that the observed reduction in insulin secretion is likely due to the observed altered β‐cell identity (Figure 6C,D), rather than impaired exocytotic machinery. This is supported by findings in diabetic Goto‐Kakizaki rats, where glibenclamide treatment failed to improve glucose‐induced first‐phase insulin secretion, despite no observable defects in insulin granule docking or fusion events [59]. In summary, glibenclamide treatment reduces insulin secretion in vivo, either through direct inhibition on K_ATP_ channels and/or via a complex interplay of paracrine signaling within the islet.

The chronic in vivo effect of glibenclamide, increased islet hypertrophy (Figure 5A), and enhanced β‐cell proliferation (Figure 5B) compared with KK‐Placebo mice are consistent with previous reports demonstrating that SUs stimulate β‐cell proliferation [63, 64]. The lack of α‐cell proliferation (Figure 5C), alongside augmented mRNA expression of α‐cell identity genes (Figure 6E) and increased immunolabeling of glucagon and α‐cell number (Figure 5D,G), driving enhanced α:β cell ratio (Figure 5H), and elevated glucagon content (Figure 4F) in islets from KK‐Glib mice compared with KK‐Placebo mice suggest an absence of posttranscriptional regulation of β‐cell identity genes. These findings are consistent with the upregulation of the α‐cell identity markers such as *Gcg, Arx* and *MafB* mRNA [65], as well as increased glucagon positive cells [39, 66] observed in islets from high‐fat‐fed SUR1 knockout (KO) mice compared with controls. A slightly lower proportion of α‐cells has been reported in young SUR1KO mice compared with controls, but this proportion increases with age, accompanied by a rise in pancreatic glucagon content [67]. In contrast, diabetic rats (streptozotocin‐induced) treated with glibenclamide for 24 weeks showed no differences in the total volume of β‐ or α‐cells compared with untreated diabetic rats [68]. Similarly, the lack of differences in *Sst* mRNA levels (Figure 6F), δ‐cell number (Figure 5G), and Sst‐positive cells (Figure 5E) in islets from KK‐Glib and KK‐Placebo mice aligns with the comparable proportion of δ‐cells observed in adult SUR1KO islets relative to age‐matched controls [69].

**FIGURE 7:**
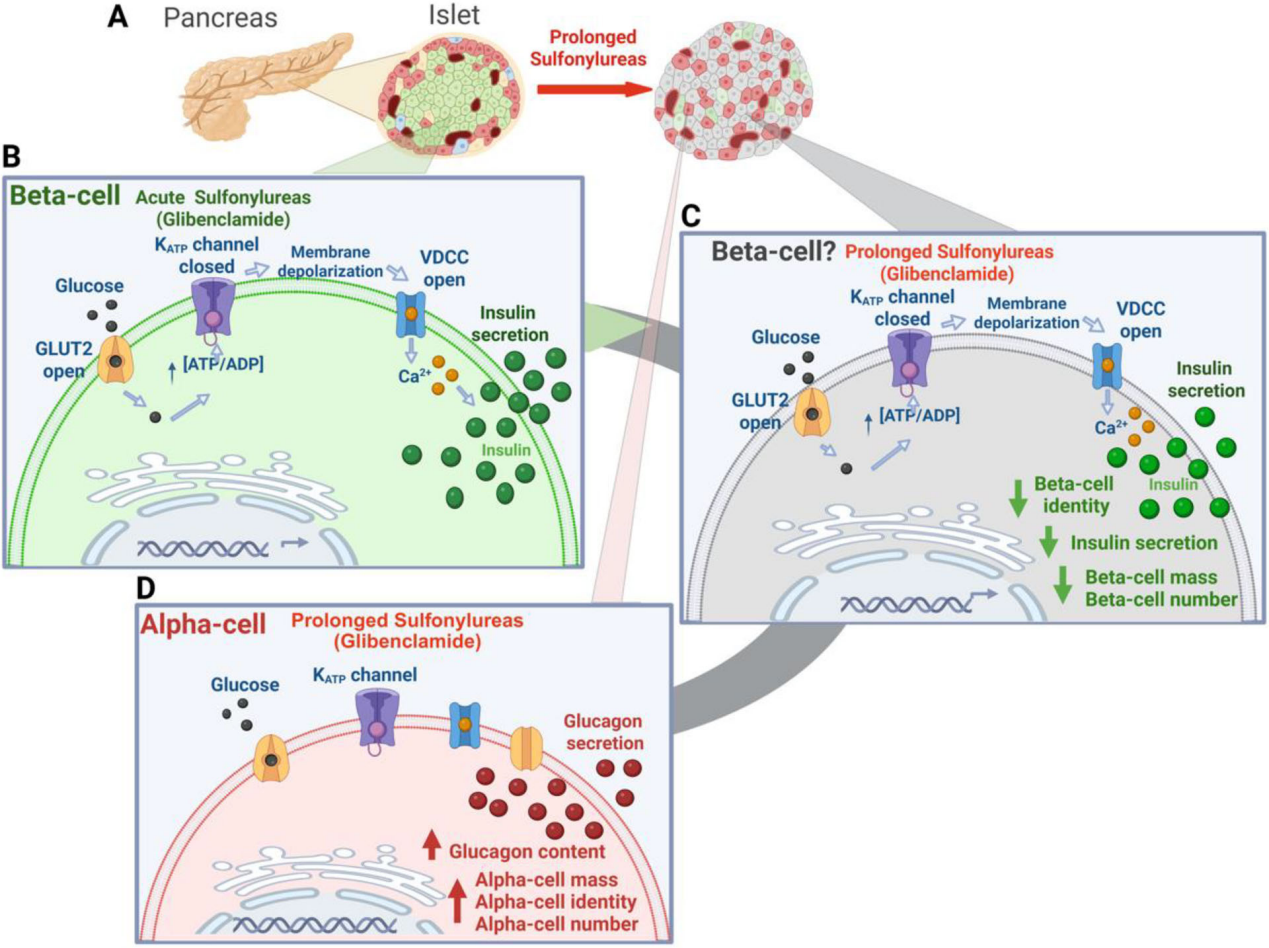
Schematic illustration of the proposed mechanism leading to secondary β‐cell failure in response to chronic sulfonylurea treatment. (A) Murine islet cell composition: beta‐cells (green), alpha‐cells (dark red), delta‐cells (blue), and polypeptide‐cells (yellow); and changes in islet architecture and cell composition upon prolonged sulfonylurea (glibenclamide) therapy in vivo. (B) Mechanism of insulin secretion in pancreatic beta‐cells upon glibenclamide treatment. (C) Loss of mature beta‐cell identity and lack of insulin secretion following prolonged glibenclamide therapy. (D) Consequential increased alpha‐cell identity and glucagon content upon prolonged glibenclamide therapy. The back arrow denotes secondary failure of beta‐cells and illustrates the alterations in alpha‐ and beta‐cell populations associated with prolonged sulfonylurea therapy in diabetes. Created in https://biorender.com.

Although mRNA levels of key β‐cell identity markers—*Ins1*, *Nkx6.1*, *Pdx1*, and *MafA—*did not differ between islets from KK‐Glib and KK‐Placebo mice (Figure 6A), immunostaining of NKX6.1 and PDX1 revealed a significant reduction in β‐cells from KK‐Glib compared with KK‐Placebo mice (Figures 6C,D), indicating loss of mature β‐cell identity. These results correlate with a recent report demonstrating decreased β‐cell identity in human islets chronically incubated with glibenclamide [70]. This finding is accompanied by a decrease in β‐cell mass (Figure 5F). These results align with unchanged *Pdx1, Nkx6.1*, and *MafA* mRNA levels in SUR1KO β‐cells [69]. The increased β‐cell proliferation alongside reduced maturity supports the prevailing view that mature, secretory β‐cells lack proliferative capacity [71, 72]. Together, these results suggest that SUs disrupt β‐cell maturity within the islet, possibly resulting in a coexistence of both mature, secretory and immature, proliferative β‐cell populations. Our results showing loss of β‐cell identity in islets from KK‐Glib‐treated mice closely align with a recent report that chronic exposure of human islets to glibenclamide for 4–7 days induces β‐cell identity loss, ER stress and impaired β‐cell function with increased insulin secretion at low glucose and reduced GSIS [70]. Despite the loss of β‐cell identity (Figure 6C,D) in islets from KK‐Glib mice compared with KK‐Placebo mice, insulin content was significantly increased (Figure 4C). This increase may be partly attributed to β‐cell proliferation (Figure 5B) and the elevated *Ngn3* mRNA levels (Figure 6B) and a potential enhancement in β‐ or α‐cell neogenesis. *Ngn3* is essential for establishing an endocrine progenitor state that promotes differentiation into the various islet cell subtypes [73]. These findings are consistent with the upregulation of *Ngn3* mRNA levels in β‐cells from SUR1 KO mice [65], as well as with increased NGN3 positive cells, elevated *Gcg, Arx* and *Pou3f4* mRNA levels, and a significant increase in α‐cell number in the developing rat pancreata upon glibenclamide treatment [74]. However, it is noteworthy that chronic glibenclamide treatment in rats induces degranulation of a subpopulation of β‐cells that exhibit increased insulin synthesis through enhanced translation [75, 76]. This may partially explain unchanged mRNA levels of β‐cell identity markers (Figure 6A) alongside increased insulin content (Figure 4C) and reduced PDX1 and NKX6.1 protein levels (Figure 6C,D), indicating the presence of a heterogenous β‐cell population following glibenclamide treatment. Additionally, long‐term glibenclamide treatment in KK mice shows notable parallels with SUR1KO mice, including increase expression of α‐cell identity genes and Ngn3 [65], impaired glucagon secretion [56], unaltered insulin sensitivity [77, 78, 79], and altered islet architecture [39, 56, 69]. Moreover, despite mild glucose intolerance, SUR1KO mice exhibit impaired insulin secretion in response to glucose [80, 81], a defect also observed in glibenclamide‐treated KK mice. In summary, we demonstrate that chronic in vivo glibenclamide treatment alters islet cell identity, characterized by decreased β‐cell identity, increased α‐cell number and identity, and elevated glucagon content, accompanied by upregulation of *Ngn3* (Figure 7). These combined changes likely contribute to sulfonylurea failure in maintaining glucose homeostasis.

## 4 Conclusion

We demonstrate that sulfonylurea treatment in polygenic diabetic KK mice is also prone to secondary failure. Insulin secretion is impaired following glibenclamide treatment, with no improvement in glycemia compared with KK‐Placebo mice. However, one limitation of the study is that we could not determine whether glibenclamide increases Sst secretion by inhibiting K_ATP_ channels in δ‐cells, thereby suppressing insulin secretion via paracrine signaling. Additionally, glibenclamide improved insulin sensitivity during the early phase of the treatment, but this effect was lost in the long‐term administration, suggesting an initial enhancement of peripheral glucose sensitivity. Surprisingly, glibenclamide treatment also increased *Ngn3* mRNA levels, indicative of expanded endocrine progenitor cells, along with increased α‐cell identity markers (*Gcg, Arx, MafB*, and *Irx2*), suggesting alterations in islet cell identity. Unfortunately, we were unable to measure NGN3 protein levels due to the lack of suitable primary antibodies. Future studies using mouse models with linage‐tracing capabilities could help determine whether SUs induce β‐ to α‐cell transdifferentiation, which remains a limitation of our study. Thus, glibenclamide may contribute to the secondary failure of SUs by accelerating the decline of functional β‐cell mass in individuals with T2D. Therefore, the use of long‐term SUs as a monotherapy for T2D should be approached with caution, given concerns about their long‐term efficacy demonstrated in human studies.

## 5 Materials and Methods

### 5.1 Animals and Glibenclamide Pellet Implantation

All procedures were approved and carried out in compliance with the institutional animal care and use committee (IACUC, Protocol #22‐0435) of Washington University in St. Louis. Five‐week‐old male KK (homozygous for A<y>, #002468) mice were purchased from the Jackson Laboratory. Mice were housed in individual cages and acclimatized to constant temperature (23 ± 2°C) on a 12‐h light/dark cycle on a standard chow diet (Picolab Rodent Diet20, #5053). At 6 weeks of age, mice were randomly divided into two groups: (i) implanted with 2.5 mg glibenclamide, 60‐day slow‐release pellets or (ii) placebo pellets (Innovative Research of America, Sarasota/FL). Mice were anesthetized with tribromoethanol (Avertin; 0.25 mg/g mouse BW) and pellets implanted under the skin of the neck using a trocar as described previously [30]. Body weights and nonfasted blood glucose were measured at noon 5 days a week. Nonfasted blood glucose was measured via tail vein incision utilizing a Bayer Contour T5 glucometer (Mishiwaka, IN). At Day 48 postimplantation, mice were sacrificed on a fed state and blood, pancreata, islets, and tissues were collected.

### 5.2 Glucose and Insulin Tolerance Tests

For glucose tolerance tests (GTTs), mice were intraperitoneally (i.p.) injected with d‐glucose (2 g/kg BW) after overnight (16 h) fasting and the experiment performed at 9AM. Blood glucose was measured before (0 min) and post‐i.p. (15, 30, 45, 60, 90, and 120 min) via tail vein utilizing a Bayer‐Contour T5 glucometer (Mishiwaka, IN). During the GTT, blood was collected in heparinized tubes at time 0 and 30 min post‐i.p. glucose injection for measurement of plasma insulin and glucagon. For ITTs mice were injected with 1.0 U/kg of human insulin after 5 h morning fasting, with the experiment performed at 1PM. *K*_ITT_ (the rate of blood glucose decline after insulin injection) was calculated by the formula (0.0693/*t*_1/2_) × 100 and expressed in %/min [82].

### 5.3 Refeeding Experiment

Mice were fasted overnight (16 h) and chow diet was reintroduced back to allow mice feed freely for 2 h. Blood glucose, body weight, and insulin and glucagon levels (blood collected in heparinized tubes) were measured before (0 min) and after refeeding (120 min).

### 5.4 Body Composition Analysis

Body composition (lean and fat mass) analysis was performed in unanesthetized mice on a fed state using an EchoMRI 3‐1 device (Echo Medical Systems) via the Washington University Diabetic Mouse Models Phenotyping Core Facility.

### 5.5 Indirect Calorimetry

All comprehensive metabolic, behavioral, and physiological measurements were performed in a PhenoMaster System (TSE systems) via the Washington University Diabetic Mouse Models Phenotyping Core Facility. Mice on a fed state were placed at room temperature in separate chambers of the PhenoMaster open‐circuit calorimetry, provided with food and water ad libitum, and allowed to acclimatize for 12 h. Indirect calorimetry (VO_2_, VCO_2_), RER, food intake, heat, and movement were measured for at least 24 h for a minimum of one light cycle (6:01 a.m. to 6:00 p.m.) and one dark cycle (6:01 p.m. to 6:00 a.m.).

### 5.6 Plasma Hormone Measurements

Insulin and glucagon levels during ipGTT and refeeding experiments were measured utilizing insulin and glucagon ELISA kits, Crystal Chem (#90080, #81858).

### 5.7 Plasma Lipid Measurements

Mice were fasted for 4 h and blood was drawn from tail vein for plasma lipid (triglyceride, cholesterol, and FFA) measurement at the Washington University Diabetes Models Phenotyping Core (https://diabetesresearchcenter.dom.wustl.edu/diabetes‐models‐phenotyping‐core).

### 5.8 Islet Isolation

Mouse pancreata were cannulated through the common bile duct and perifused with Collagenase solution (0.45 mg/mL Type XI; Sigma Corp, St. Louis, MO), removed and digested at 37°C for 10 min and washed three times in cold Hank's solution. Islets were hand‐picked under a dissecting microscope, cultured for 1 h or overnight in RPMI complete media (11 mM glucose) supplemented with 10% FBS, 100 units/mL penicillin, and 100 µg/mL streptomycin (Thermo Fisher Scientific) for GSIS or frozen down immediately for qRTPCR.

### 5.9 Glucose‐Stimulated Insulin and Glucagon Secretion (GSIS and Glucose‐Stimulated Glucagon Secretion)

Freshly isolated or cultured overnight islets, were incubated in low glucose (2.8 mM glucose in Krebs‐Ringer buffer + 0.1%BSA) for 1 h for stabilization. Groups of 10 islets were incubated in 2.8 or 16.7 mM glucose, or in the presence of 30 mM KCl for 1 h at 37°C (two‐three technical replicates per sample). Samples were then centrifuged and supernatant collected for GSIS and GSGS. Islets were retrieved and lysed in 0.2 N/80% acid‐ethanol for determination of total insulin and glucagon content. Insulin and glucagon concentrations were determined using ELISA kits (Crystal Chem#90080, #81858, respectively).

### 5.10 Immunohistochemical and Morphometric Analysis

Pancreata from both groups were collected at Day 48 posttreatment and fixed in 10% formalin and paraffin embedded for sectioning. Serial sections of 5 µm, 100 µm apart, and H&E staining were performed at the Anatomic/Molecular Pathology Core, Washington University. For immunohistochemical staining, slides were deparaffinized, rehydrated, and underwent antigen retrieval with sodium citrate. Then blocked in 3% bovine serum albumin and incubated overnight in primary antibodies against insulin (1:100; Cell Signaling Technology; cat# 3014), glucagon (1:100; Abcam; cat# Ab10988), Sst (1:200; Proteintech; cat# 17512‐1‐AP), Nkx6.1 (1:400; Novus Biological; cat# NBP1‐49672), Pdx1 (1:500; DSHB; Cat#F6A11), and Ki67 (1:500; Abcam; cat# ab15580). This was followed by incubation in secondary antibodies goat anti‐rabbit AF‐488 or AF‐594 (1:500; Invitrogen; cat# A21206, A11012) and goat anti‐mouse AF‐488 or AF‐594 (1:500; Invitrogen; cat# A21202, A21203) and mounted using anti‐fade media with DAPI. At least four pancreatic sections from 3 to 5 mice of each genotype were covered systematically by accumulating images from 8 to 10 islets/pancreatic section from nonoverlapping fields on an inverted EXC‐500 fluorescent microscope (Visual Dynamix, Chesterfield, MO). Images were quantified using ImageJ 1.52q software (NIH, Bethesda, MD). α, β, and δ cell numbers were calculated per islet. β‐ and α‐cell mass was determined by multiplying the pancreatic weight by the proportion of the insulin‐positive or glucagon‐positive area relative to total pancreatic area. The percentage of NKX6.1^+^INS^+^, PDX1^+^INS^+^, and Ki67^+^INS^+^ cells was determined per INS^+^ cells.

### 5.11 RNA Isolation and Quantitative Real‐Time PCR

Total RNA from islets were extracted using RNeasy Mini Kit (Qiagen, Germantown, MD) and reverse transcribed using the High‐Capacity cDNA Reverse Transcription Kit (Applied Biosystems, Waltham, MA). Real‐time quantitative PCR was performed using Fast SYBR Green Master Mix (Applied Biosystems) on a Step‐One Plus Real‐Time PCR System (Applied Biosystems). Relative gene expression (normalized mL32 mRNA levels) was calculated using the comparative Ct method formula 2^−ΔΔCt^. The primer sequences are provided in Table S1.

### 5.12 Statistical Analyses

Data were analyzed by GraphPad Prism 10.0 (GraphPad Software, La Jolla, CA) and is presented as mean ± standard error of mean (SEM). The significant differences between groups was evaluated by two‐tailed unpaired *t*‐test or by two‐way ANOVA followed by posthoc Tukey's test for multiple comparisons, *p* < 0.05 was considered statistically significant.

## Author Contributions

Sumit Patel: writing – original draft, conceptualization, visualization, validation, methodology, investigation, and formal analysis. Part of this work was included in Sumit Patel's doctoral thesis at Washington University in St. Louis. Zihan Yan: investigation and formal analysis. Maria S. Remedi: writing – review and editing, conceptualization, investigation, supervision, funding acquisition, and formal analysis. All authors have read and approved the final manuscript.

## Funding

This work was supported by National Institutes of Health grants R01DK123163 and R01DK133838 (to M.S.R.). The authors also acknowledge the Diabetes Models Phenotyping Core and the Metabolic Tissue Function Core, Diabetes Research Center, Washington University in St. Louis, MO, National Institutes of Health grants P30 DK020579 and P30 DK056341 (Nutrition Obesity Research Center). The funders had no role in the study design, data collection and analysis, decision to publish, or preparation of the manuscript.

## Ethics Statement

All procedures were approved and carried out in compliance with the institutional animal care and use committee (IACUC, Protocol #22‐0435 to MSR) of Washington University in St. Louis.

## Conflicts of Interest

The authors declare no conflicts of interest.

## Supporting information




**Figure S1**: Tissue weights after 48 days posttreatment. (A) Liver weights, (B) epididymal white adipose tissue (eWAT) weight, and (C) brown adipose tissue WAT (BAT) weight, normalized to BW (*n* = 6/7 mice/group). Data are mean ± SEM. Statistical analysis was conducted by unpaired Student's *t*‐test. *p < 0.05, **p < 0.01, ***p < 0.001, ****p < 0.0001. Nonsignificant differences are not shown.
**Figure S2**: Body weight changes during fasting/refeeding and GSIS after islets were cultured overnight (drug washout). Body weight at (A) Day 19 (*n *= 11–13 mice/group) and (B) Day 38 (*n *= 11–13 mice/group). (C) Quantitative real‐time PCR analysis for *Epac2* and *Snap25* (*n *= 6/7 mice/group) in islets from KK‐Placebo and KK‐Glib mice. (D) GSIS at basal (2.8 mM) glucose, high (16.7 mM) glucose, or KCl (30 mM) after islets were cultured overnight, (E) GSIS normalized to total insulin content after islets were cultured overnight, and (F) total insulin content after islets were cultured overnight (harvested 48 days postintervention, *n *= 3–4 mice/group). Data are mean ± SEM. Statistical analysis was conducted by two‐way ANOVA followed by the posthoc Tukey's test. *p < 0.05, **p < 0.01, ****p < 0.0001. Nonsignificant differences are not shown.
**Figure S3**: Representative immunostaining images of (A) glucagon (red) staining and (B) somatostatin (red) staining in pancreata after 48 days posttreatment (scale bars 100 µm, *n *= 3–4 mice/group).
**Figure S4**: (A) Representative immunostaining images of NKX6.1 (red) staining, (B) PDX1 (red) staining and (C) Ki67 (red) staining in pancreata after 48 days posttreatment (scale bars 100 µm, *n *= 3–4 mice/group).
**Table S1**.

## Data Availability

The datasets generated and/or analyzed during the current study are available within the article, supplementary data, or available from the corresponding author upon reasonable request.
