# Supplementary material for: Loss of Beta‐Cell Identity and Function as a Mechanism of Secondary Failure of Sulfonylurea Therapy in Diabetes
Source: MedComm (2020). 2026 Feb 9;7(2):e70588. doi: 10.1002/mco2.70588 (PMC12887434; doi:10.1002/mco2.70588)
Supplement: Supplementary file 1 — Figure S1: Tissue weights after 48 days posttreatment. (A) Liver weights, (B) epididymal white adipose tissue (eWAT) weight, and (C) brown adipose tissue WAT (BAT) weight, normalized to BW (n = 6/7 mice/group). Data are mean ± SEM. Statistical analysis was conducted by unpaired Student's t‐test. *p < 0.05, **p < 0.01, ***p < 0.001, ****p < 0.0001. Nonsignificant differences are not shown. Figure S2: Body weight changes during fasting/refeeding and GSIS after islets were cultured overnight (drug washout). Body weight at (A) Day 19 (n = 11–13 mice/group) and (B) Day 38 (n = 11–13 mice/group). (C) Quantitative real‐time PCR analysis for Epac2 and Snap25 (n = 6/7 mice/group) in islets from KK‐Placebo and KK‐Glib mice. (D) GSIS at basal (2.8 mM) glucose, high (16.7 mM) glucose, or KCl (30 mM) after islets were cultured overnight, (E) GSIS normalized to total insulin content after islets were cultured overnight, and (F) total insulin content after islets were cultured overnight (harvested 48 days postintervention, n = 3–4 mice/group). Data are mean ± SEM. Statistical analysis was conducted by two‐way ANOVA followed by the posthoc Tukey's test. *p < 0.05, **p < 0.01, ****p < 0.0001. Nonsignificant differences are not shown. Figure S3: Representative immunostaining images of (A) glucagon (red) staining and (B) somatostatin (red) staining in pancreata after 48 days posttreatment (scale bars 100 µm, n = 3–4 mice/group). Figure S4: (A) Representative immunostaining images of NKX6.1 (red) staining, (B) PDX1 (red) staining and (C) Ki67 (red) staining in pancreata after 48 days posttreatment (scale bars 100 µm, n = 3–4 mice/group). Table S1. [file MCO2-7-e70588-s001.pdf]

Loss of Beta-cell Identity and Function as a Mechanism of Secondary Failure of Sulfonylurea Therapy in Diabetes

Sumit Patel[1], Zihan Yan[1] and Maria S. Remedi[1,2,3]*

[1]Department of Medicine, Division of Endocrinology, Metabolism and Lipid Research, Washington University in St Louis, School of Medicine, 660 South Euclid Avenue, St Louis, MO. USA

[2]Deparment of Cell Biology and Physiology, Washington University in St Louis, School of Medicine, 660 South Euclid Avenue. St Louis, MO. USA.

[3]Center for the Investigation of Membrane Excitability Diseases, Washington University in St Louis, School of Medicine, 660 South Euclid Avenue. St Louis, MO. USA

*Address all correspondence and reprint requests to MSR: e-mail: mremedi@wustl.edu

Table S1

**Gene**		**5'--> 3'**
Aldh1a3	Forward	AGGCTGTATTAAGACCTTCAG
	Reverse	GGAAGTTCCATGGTGTAATG
Nkx6.1	Forward	AGAGAGCAGGCTTGGCCTATTC
	Reverse	GTCGTCAGAGTTCGGGTCCAG
Gcg	Forward	CATTCACCAGCGACTACAGCAA
	Reverse	TCATCAACCACTGCACAAAATCT
MafB	Forward	AGGACCTGTACTGGATGGC
	Reverse	CACTACGGAAGCCGTCGAAG
Pdx1	Forward	GGAAGAGCCCAACCGCGTCC
	Reverse	GGCGGGGCCGGGAGATGTAT
Ins1	Forward	CAGCAAGCAGGTCATTGTTT
	Reverse	GGGACCACAAAGATGCTGTT
MafA	Forward	CTCCTCCAAGCGCACGTGGT
	Reverse	TTCAGCAAGGAGGAGGTCAT
Kir6.2	Forward	CGG GCG CAT GGT GAC AGA GG
	Reverse	CGA TGG GCC TGG GCC GTT TT
Sur1	Forward	TGA GCA TTG GAA GAC CCT CAT
	Reverse	CAG CAC CGA AGA TAA GTT GTC A
Arx	Forward	TTCCAGAAGACGCACTACCC
	Reverse	TCTGTCAGGTCCAGCCTCAT
Irx2	Forward	ACGCACACCACCGGAATG
	Reverse	ATGGATAGGCCGCACTGC
mL32	Forward	TTCCTGGTCCACAATGTCAA
	Reverse	GGCTTTTCGGTTCTTAGAGGA
Ngn3	Forward	TCTCAAGCATCTCGCCTCTTC
	Reverse	ACAGCAAGGGTACCGATGAGA
Sst	Forward	ATGCTGTCCTGCCGTCTCCA
	Reverse	CTAACAGGATGTGAATGTCTTCCAG
Slc7a2	Forward	TCTATGTTCCCCTTACCCCGA
	Reverse	TGACTGCCTCTTACTCACTCTT
Rfx6	Forward	GCTTGCTGGTCTACCCTGAG
	Reverse	TGCCGTTGTTTAACTGCATTTC
Isl1	Forward	ATGATGGTGGTTTACAGGCTAAC
	Reverse	TCGATGCTACTTCACTGCCAG
Epac2	Forward	CAAGATGTCTTGGTACTGGAGAAG
	Reverse	CAGGTGTTCCTGACATCACAGTAT
Snap25	Forward	CAACTGGAACGCATTGAGGAA
	Reverse	GGCCACTACTCCATCCTGATTAT

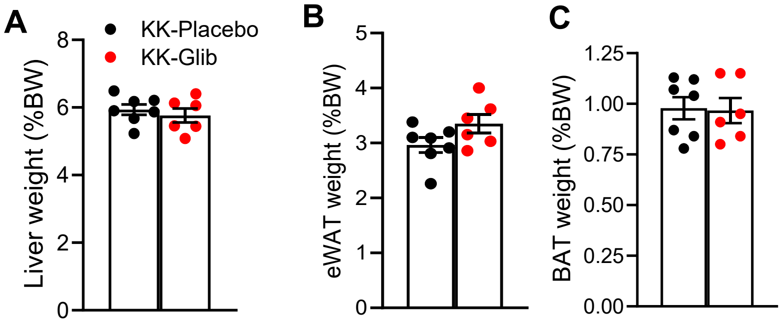

Figure S1. ***Tissue weights after 48 days post-treatment. A*** Liver weights, ***B*** Epididymal white adipose tissue (eWAT) weight and ***C*** Brown adipose tissue WAT (BAT) weight, normalized to BW (*n*=6/7 mice/group). Data are mean ± SEM. Statistical analysis was conducted by unpaired Student's t-test. *P < 0.05, **P < 0.01, ***P < 0.001, ****P < 0.0001. Nonsignificant differences are not shown.

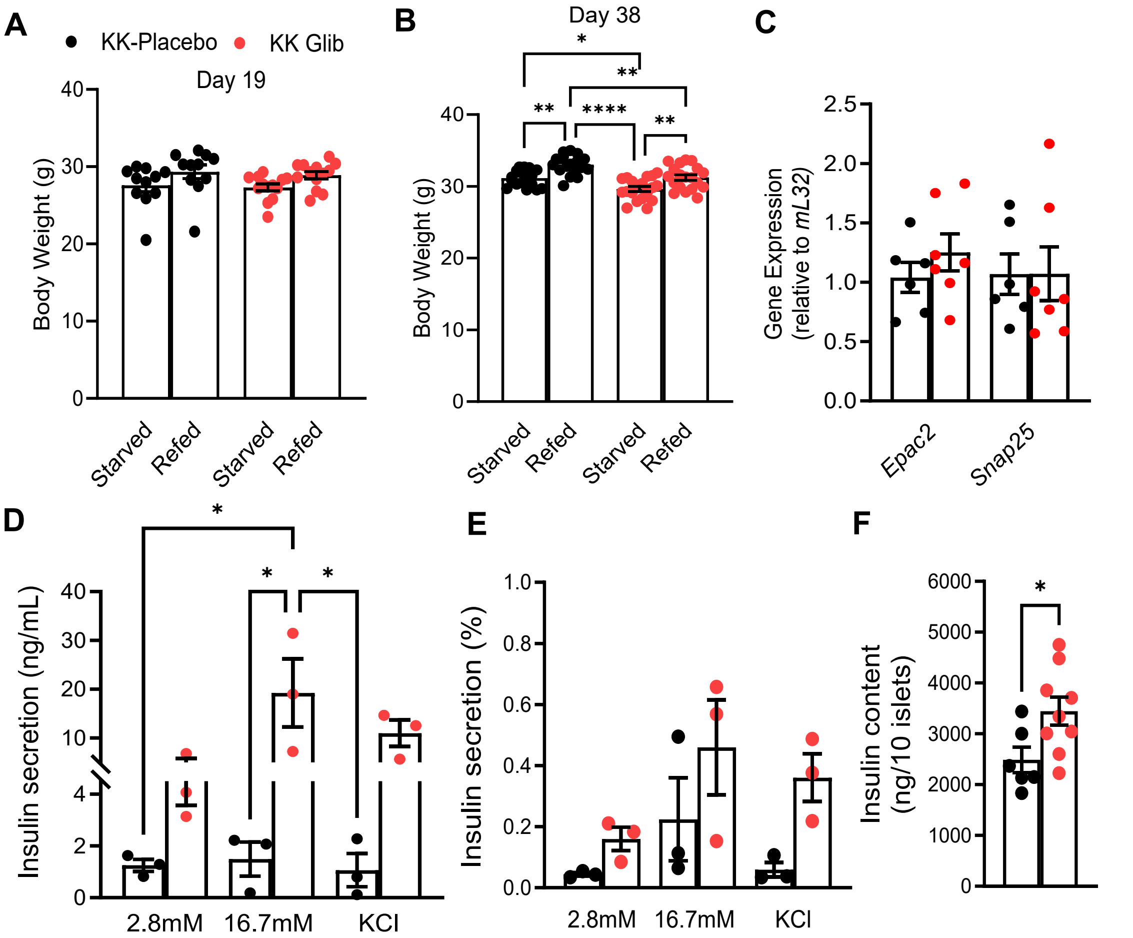

Figure S2. *Body weight changes during fasting/refeeding and GSIS after islets were cultured overnight (drug washout).* Body weight at ***A*** Day 19 (*n*=11-13 mice/group) and ***B*** Day 38 (*n*=11-13 mice/group). ***C*** Quantitative real-time PCR analysis for *Epac2* and *Snap25* (*n*=6/7 mice/group) in islets from KK-Placebo and KK-Glib mice. ***D*** GSIS at basal (2.8 mM) glucose, high (16.7 mM) glucose or KCl (30 mM) after islets were cultured overnight, ***E*** GSIS normalized to total insulin content after islets were cultured overnight and ***F*** Total insulin content after islets were cultured overnight (harvested 48 days post-intervention, *n*=3-4 mice/group). Data are mean ± SEM. Statistical analysis was conducted by two-way ANOVA followed by the post hoc Tukey's test. *P < 0.05, **P < 0.01, ****P < 0.0001. Nonsignificant differences are not shown.

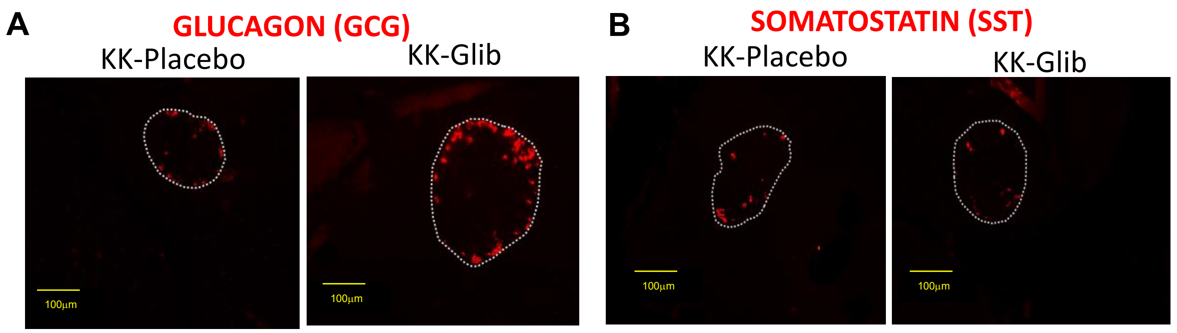

Figure S3. Representative immunostaining images of ***A*** Glucagon (red) staining and ***B*** Somatostatin (red) staining in pancreata after 48 days post-treatment (scale bars 100 μm, *n*=3-4 mice/group).

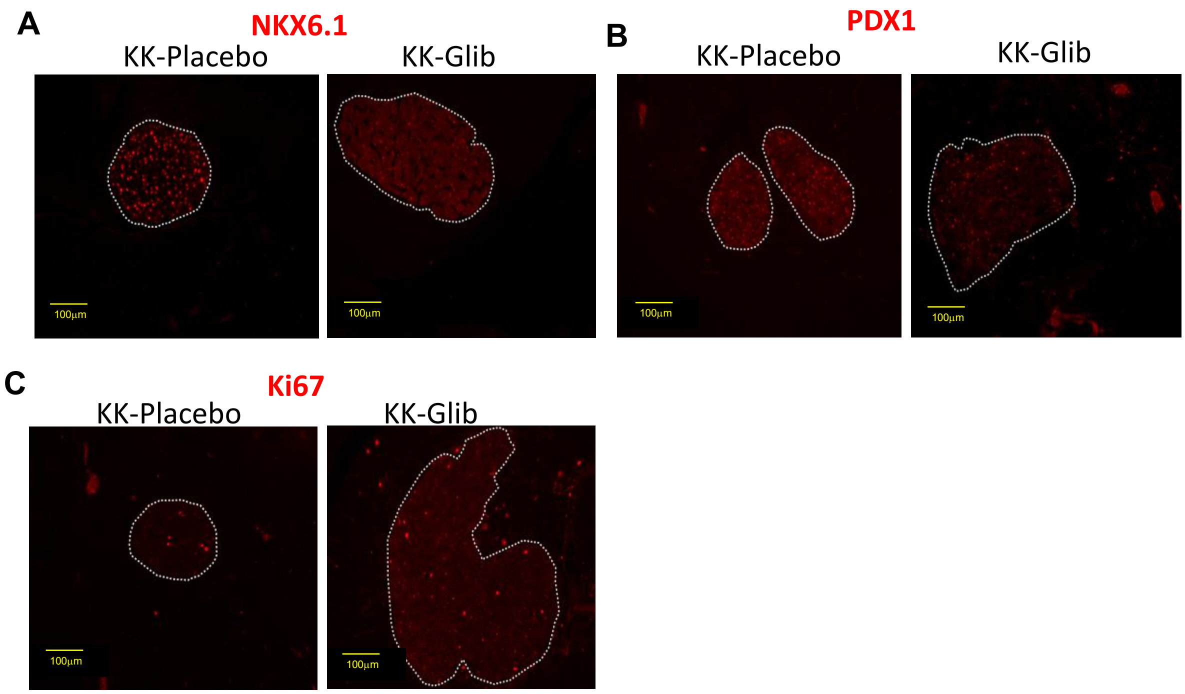

Figure S4 ***A.*** Representative immunostaining images of NKX6.1 (red) staining, ***B*** PDX1 (red) staining and ***C*** Ki67 (red) staining in pancreata after 48 days post-treatment (scale bars 100 µm, *n*=3-4 mice/group).
